# A Case of Recurrent Gestational Hypokalemia Due to an Exaggerated Physiological Response to Pregnancy: The Importance of Using Pregnancy-Specific Reference Ranges

**DOI:** 10.7759/cureus.51213

**Published:** 2023-12-28

**Authors:** Benedicta N Sarfo-Adu, Dineth Jayatilake, Samson O Oyibo

**Affiliations:** 1 Obstetrics, Peterborough City Hospital, Peterborough, GBR; 2 General Medicine, Peterborough City Hospital, Peterborough, GBR; 3 Diabetes and Endocrinology, Peterborough City Hospital, Peterborough, GBR

**Keywords:** recurrent hypokalemia, reference ranges, pregnancy-specific, pregnancy, gestational hypokalemia, hypokalemia

## Abstract

Hypokalemia in pregnancy can occur secondary to hemodilution of pregnancy, physiological changes during pregnancy, or other pathological conditions. It should be investigated the same way as in non-pregnancy with particular emphasis on the importance of using pregnancy-specific reference ranges when interpreting clinical laboratory test results. Here, we present a case of a woman who had late-trimester gestational hypokalemia requiring potassium supplementation affecting four consecutive pregnancies. We thought that there was accompanying hypomagnesemia and hypobicarbonatemia in previous pregnancies, so we suspected a form of renal tubular dysfunction exacerbated by pregnancy. Subsequent investigation and the use of pregnancy-specific reference ranges revealed that this was an exaggerated physiological response to pregnancy.

## Introduction

Hypokalemia is one of the most common electrolyte abnormalities encountered in clinical practice and is a potentially life-threatening condition [1]. Normal serum potassium levels have a narrow range between 3.5 and 5.5 mmol/L. The severity of hypokalemia is categorized as mild when the serum potassium level is 3.0-3.4 mmol/L, moderate when the serum potassium level is 2.5-3.0 mmol/L, and severe when the serum potassium level is less than 2.5 mmol/L. The clinical manifestations include muscle weakness, muscle cramps, and cardiac arrhythmias in moderate to severe cases [1]. Hypokalemia can be caused either by decreased intake of potassium or by excessive losses of potassium through the renal or the gastrointestinal tract, and therefore a methodical approach is required to diagnose the cause.

Physiological and biochemical adaptations, which lead to hemodilution, contributes to the lower laboratory results observed during pregnancy. Hence, the use of pregnancy-specific laboratory reference values is recommended [2]. The physiological hyperventilation observed in pregnancy can also give rise to mild hypokalemia secondary to mild respiratory alkalosis and compensatory transcellular electrolyte movements [3,4].

Hypokalemia in the pregnant state should be investigated the same way as in the non-pregnant state. Here, we present a case of a woman who had late-trimester gestational hypokalemia requiring potassium supplementation affecting four consecutive pregnancies.

## Case presentation

A 35-year-old woman was referred to the endocrinology department because of mild hypokalemia in pregnancy at 23 weeks of gestation. The same had occurred in her previous three pregnancies, during which time she required potassium supplementation. She had no symptoms on systemic review.

During her first pregnancy, she was discovered to have severe hypokalemia (2.6 mmol/L) at 34 weeks of gestation, which later fell to a nadir of 2.3 mmol/L while on potassium supplements. During her second pregnancy, she was discovered to have mild hypokalemia (3.4 mmol/L) at 30 weeks of gestation, which later fell to a nadir of 3.0 mmol/L while on potassium supplements. During her third pregnancy, she was discovered to have mild hypokalemia (3.3 mmol/L) at 33 weeks of gestation, which later fell to a nadir of 3.2 mmol/L despite being on potassium supplements. She also had a history of third-trimester gestational hypertension in all her previous pregnancies. Her medication list included aspirin, iron supplements, pregnancy vitamins, and labetalol (100 mg twice a day). She was not on any diuretics, proton pump inhibitors, or herbal remedies. She was a non-smoker and did not drink alcohol. On examination, she was 25 weeks pregnant. She had a heart rate of 66 beats per minute, respiratory rate of 14 breaths per minute, and a blood pressure of 130/78 mmHg on medication. She had no evidence of peripheral edema.

Investigations 

Blood investigations at 25 weeks of gestation demonstrated mild hypokalemia. Her serum bicarbonate level was below the non-pregnant reference range but within the pregnancy-specific reference range (Table 1). Blood results at 31 and 32 weeks of gestation demonstrated serum magnesium and bicarbonate levels slightly below the non-pregnant reference range but within the pregnancy-specific reference range (Table 2). Urinalysis demonstrated slight renal wasting of potassium in the presence of mild hypokalemia, and a urine pH was 7.0 in the presence of mild hypobicarbonatemia (Table 3). Serum protein electrophoresis, immunoglobulins, and complement factors were normal. A full autoimmune antibody screen was negative. Urine culture for bacterial growth was negative. In addition, the urine was negative for glucose, protein, and ketones. Subsequent arterial blood gas sampling demonstrated mild respiratory alkalosis with metabolic compensation (Table 4).

**Table 1:** Laboratory tests at 25 weeks of gestation Results indicated mild hypokalemia with a normal serum anion gap. The serum anion gap was derived from the sum of the serum sodium and potassium levels minus the sum of the serum bicarbonate and chloride levels.

Blood test	Result	Non-pregnant reference range	Second-trimester pregnancy-specific reference range
Hemoglobin (g/L)	115	100-150	97-148
White cell count (10^9^/L)	8	4.5-15.0	5.6-14.8
Platelet count (10^9^/L)	275	150-400	155-409
Sodium (mmol/L)	139	133-146	129-148
Potassium (mmol/L)	3.2	3.5-5.3	3.3-5.0
Chloride (mmol/L)	107	95-108	97-109
Bicarbonate (mmol/L)	19	22-29	18-26
Anion gap	16	4-16	10-18
Creatinine (µmol/L)	58	45-84	35-71
Urea (mmol/L)	3.2	2.5-7.8	1.1-4.6
Serum-corrected calcium (mmol/L)	2.42	2.2-2.6	2.05-2.25
Phosphate (mmol/L)	1.2	0.8-1.5	0.81-1.49
Magnesium (mmol/L)	0.8	0.8-1.0	0.63-0.92
Lactate (mmol/L)	1.2	0.6-2.5	0.6-2.5
Creatinine phosphokinase	55	25-200	25-75
Fasting glucose (mmol/L)	3.6	< 5.6	< 4.4
Thyroid stimulating hormone (mU/L)	2.96	0.3-4.2	0.37-3.6
9-am cortisol (nmol/L)	303	250-600	276-1159
Renin (mU/L)	12.0	5.4-30	-
Aldosterone (pmol/L)	104	90-405	250-2885
Bilirubin (µmol/L)	3	< 21	< 13
Alanine transferase (U/L)	10	< 33	< 33
Alkaline phosphatase (U/L)	104	30-130	25-125
Total protein (g/L)	66	60-80	57-69
Albumin (g/L)	38	35-50	26-45
Globulin (g/L)	27	20-35	-
Parathyroid hormone (pmol/L)	4	1.6-6.9	1.9-2.7
Angiotensin converting enzyme (U/L)	58	20-70	1-36

**Table 2:** Repeat blood results at 31, 32, and 34 weeks of gestation Potassium supplements dose increments depending on serum potassium levels. The serum magnesium and bicarbonate levels were lower than the non-pregnant reference range, but within the pregnancy-specific reference range.

Blood tests	Result at each gestation			Third-trimester pregnancy-specific reference range
	31 weeks	32 weeks	34 weeks	
Potassium (mmol/L)	3.2	3.8	3.0	3.3-5.1
Magnesium (mmol/L)	0.69	0.66	-	0.46-0.92
Bicarbonate (mmol/L)	20	20	20	18-26
Potassium supplement dose	Increased to two tablets twice a day	Remained on two tablets twice a day	Increased to two tablets three times a day	-

**Table 3:** Urinalysis results Results indicated slight renal wasting of potassium in the presence of mild hypokalemia. The urine anion gap was derived from the sum of the spot urine sodium and urine potassium levels minus the urine chloride level. The value was positive, indicating reduced urinary output of acid (hydrogen ions) or increased urinary output of base (bicarbonate ions) or both.

Urine test	Result	Expected range
Urine anion gap (mmol/L)	28	0-10
Urine pH	7.0	4.5-7.8
Specific gravity	1.015	1.002-1.020
Spot urine potassium (mmol/L)	17	< 15
Fractional excretion of potassium (%)	9.27	< 9.0
Potassium-creatinine ratio	5.11	< 2.5
Protein-creatinine ratio (mg/mmol)	11	< 30

**Table 4:** Arterial blood gas analysis Results indicated mild hyperventilation with mild respiratory alkalosis appropriate for pregnancy. PCO_2_ = partial pressure of carbon dioxide; PO_2_ = partial pressure of oxygen

Arterial blood gas	Result	Non-pregnant reference range	Pregnancy-specific reference range
pH	7.474	7.35-7.45	7.40-7.47
PCO_2_ (kPa)	3.82	4.27-6.40	3.70-4.20
PO_2_ (kPa)	14.83	11.07-14.40	11.07-14.40
Bicarbonate (mmol/L)	20.6	23-30	18-21
Lactate (mmol/L)	0.76	0.2-1.8	< 2.0
Base excess	-2	-2-+2	-2-+2

Historical results were reassessed using pregnancy-specific reference ranges. Results indicated chronic hypokalemia since discovery in her first pregnancy seven years before this presentation (only one normal potassium result in between the first and second pregnancies and one normal result in between the second and third pregnancies, but no potassium measurement between the third and fourth pregnancies). The hypokalemia was most severe during the first pregnancy, moderate during the second pregnancy, and mild during the third pregnancy. Intermittent magnesium, chloride, and bicarbonate checks indicated levels within the pregnancy-specific reference ranges. Her kidney function and urinary protein-creatinine ratio assessments had been normal throughout all her pregnancies.

Treatment 

Keeping in mind her previous severe hypokalemia at 34 weeks of gestation, she was commenced on potassium tablets prophylactically to prevent severe hypokalemia in her second and third pregnancies. This practice was carried into her fourth pregnancy: the patient was commenced on potassium tablets (one twice a day) at 27 weeks of gestation, and the dose was increased to two tablets twice a day at 31 weeks gestation and again increased to two tablets three times a day at 34 weeks of gestation. Each potassium tablet (Sandoz-K®) contains 470 mg (12 mmol) of potassium.

Outcome and follow-up

Serum potassium levels were normal at 35 weeks of gestation and remained stable until delivery without any severe drop in levels. Potassium supplementation was stopped soon after delivery, and a repeat blood test done six weeks post-delivery demonstrated normal serum potassium, magnesium, and bicarbonate levels. Subsequent repeat arterial blood gas sampling demonstrated completely normal results, indicating no more hyperventilation or respiratory alkalosis.

## Discussion

We have presented a lady who has had gestational hypokalemia affecting four consecutive pregnancies and requiring potassium supplementation each time. During her fourth pregnancy, there was additional evidence of mild renal wasting of potassium. She had a positive urine anion gap, which indicated either reduced hydrogen ion (ammonia) output or increased bicarbonate output or both. We initially thought she had periods of hypomagnesemia and hypobicarbonatemia accompanying the hypokalemia in previous pregnancies, but that was not the case when pregnancy-specific reference ranges were applied. Subsequent arterial blood gas sampling and the use of pregnancy-specific reference ranges later revealed that this was a case of mild gestational respiratory alkalosis secondary to the physiological hyperventilation of pregnancy, accompanied by metabolic compensation.

Respiratory alkalosis due to hyperventilation in pregnancy is a normal physiological response to pregnancy [3,4]. Hormonal and mechanical changes contribute to changes in the pulmonary function. The tidal volume rather than the breathing rate is increased, resulting in an increased minute ventilation (by up to 48%) in pregnancy. This can be exacerbated by high circulating progesterone levels. Hyperventilation results in reduced arterial carbon dioxide tension, increased arterial oxygen tension, and chronic respiratory alkalosis [3,4]. This is accompanied by renal compensation by increasing renal excretion of bicarbonate, and the transcellular shift and conservation of hydrogen ions in exchange for potassium ions. The resultant hypokalemia is usually mild and asymptomatic. However, there is a case report describing a patient who had severe hypokalemia-induced paralysis in pregnancy [5].

There is a wide differential diagnosis for the causes of hypokalemia in pregnancy. Hypokalemia is a common finding during vomiting or hyperemesis of pregnancy [6]. However, our patient did not have such symptoms during any of her pregnancies. The use of large amounts of licorice in root form or confectionaries can also cause pseudohyperaldosteronism with hypertension, hypokalemia, and metabolic alkalosis [7]. Similarly, the use of diuretics in pregnancy has been shown to cause hypokalemia plus other electrolyte disturbances, but our patient was not on any diuretic therapy during any of her pregnancies. Distal and proximal types of renal tubular acidosis are associated with non-gap hyperchloremic hypokalemic acidosis. Renal tubular acidosis can be genetic or secondary to autoimmune disorders. There is failure to acidify the urine in the presence of metabolic acidosis [8]. Our initial thoughts were whether our patient had a primary metabolic acidosis (low serum bicarbonate) with raised urinary pH characteristic of renal tubular acidosis, but arterial blood gas sampling disproved this and revealed a metabolic compensation for an underlying mild physiological respiratory alkalosis of pregnancy.

Rare syndromes involving genetic defects in the sodium-potassium-chloride co-transporters in the renal collecting tubules (e.g., Bartter, Gitelman, and Liddle syndromes) have been associated with severe hypokalemia and metabolic alkalosis. These conditions have associated syndromic features and positive family histories [9-11]. Our patient did not have metabolic alkalosis or any syndromic features associated with these rare conditions. Cushing syndrome is also associated with hypokalemia, but our patient did not have any of the classical signs of hypercortisolemia [12]. Geller syndrome is a rare genetic disease caused by a mutation in the mineralocorticoid receptor with a resultant gain of function. Normally, the mineralocorticoid receptor is activated by aldosterone and inhibited by progesterone. In Geller syndrome, progesterone activates the mineralocorticoid receptor resulting in hypertension, hypokalemia, and metabolic alkalosis presenting during pregnancy. Patients with Geller syndrome have low renin and aldosterone levels, as opposed to high aldosterone levels found in patients with primary hyperaldosteronism [13]. Finally, primary hyperaldosteronism should also be considered in pregnant patients with severe hypertension with or without hypokalemia [14]. Although our patient did have third-trimester gestational hypertension, her serum renin and aldosterone levels were normal, and she did not have metabolic alkalosis.

In a review article, it was noted that the importance of pregnancy-specific reference ranges for laboratory tests in pregnancy is highly underappreciated and that a significant number of clinical laboratories continue to use non-pregnant reference ranges for test results in pregnancy [15]. The process of providing trimester-specific reference ranges needs to keep up with updated analytical platforms; otherwise, the standard of clinical laboratory service for maternal-fetal healthcare remains compromised [15]. The lack of understanding of the physiological response to pregnancy and not using pregnancy-specific reference ranges when interpreting laboratory test results led to misinterpretation and the over-investigation of hypokalemia in this pregnant patient.

## Conclusions

Hypokalemia is one of the most common electrolyte abnormalities encountered in clinical practice and has a wide differential diagnosis. We have described a case of recurrent gestational hypokalemia affecting four consecutive pregnancies in a young woman due to an exaggerated physiological response to pregnancy.

This case not only reminds us of the normal physiological response to pregnancy but also emphasizes the importance of using pregnancy-specific reference ranges when interpreting clinical laboratory test results in pregnancy.
